# Incidence of Bacterial Colonization of Intravenous Non-Permanent Venous Catheters in Hospitalized Equine Patients

**DOI:** 10.3390/vetsci12090788

**Published:** 2025-08-22

**Authors:** Valentina Vitale, Francesca Bindi, Fabrizio Bertelloni, Giulia Sala, Dania Cingottini, Francesca Bonelli, Micaela Sgorbini

**Affiliations:** 1 Departamento de Medicina y Cirugía Animal, Universidad CEU-Cardenal Herrera, CEU Universities, Alfara del Patriarca, 46115 Valencia, Spain; valentina.vitale@uchceu.es; 2 Department of Veterinary Sciences, University of Pisa, Viale delle Piagge 2, 56124 Pisa, Italy; fabrizio.bertelloni@unipi.it (F.B.); giulia.sala@unipi.it (G.S.); dania.cingottini@phd.unipi.it (D.C.); francesca.bonelli@unipi.it (F.B.); micaela.sgorbini@unipi.it (M.S.)

**Keywords:** horse, catheter-associated infections, risk factors

## Abstract

Bacterial contamination of intravenous catheters in hospitalized horses may pose risks for both patient care and public health due to potential antimicrobial resistance and zoonotic transmission. This prospective clinical study aimed to evaluate the incidence of catheter contamination in equine patients admitted to a Veterinary Teaching Hospital between 2020 and 2021. All horses requiring intravenous catheterization were included, and data were collected on patient signalment, clinical status, duration of catheterization, treatments, and outcomes. Two catheter types were used: 5 cm polytetrafluoroethylene and 11 cm polyurethane, both 14-gauge. Catheters were aseptically removed, and the distal ends were cultured on Blood Agar and Brain Heart Infusion broth. Bacterial isolates were identified using standard microbiological techniques, and contamination was defined as any detectable bacterial growth. Of the 58 catheters collected from 52 patients, 38 (65.5%) showed bacterial growth, predominantly *Staphylococcus* spp. Multivariable logistic regression analysis identified antibiotic treatment as significantly associated with positive bacteriological culture. These findings indicate a higher contamination rate than previously reported in horses. While colonization may not always result in clinical infection, the observed frequency of contamination underscores the need for careful catheter management and further investigation into its implications for antimicrobial stewardship.

## Simple Summary

In hospitalized horses, intravenous catheters are often needed to give fluids or medications, but they can become contaminated with bacteria, increasing the risk of infection and antibiotic resistance. This study examined how often catheter contamination occurs and what factors are involved. Over two years, 58 catheters from 52 horses were tested after removal. Bacteria were found on nearly two-thirds of them, with the most common types normally found on the skin. Horses that received antibiotics were more likely to have contaminated catheters. While not all contaminated catheters cause illness, the high rate is concerning and highlights the need for better catheter care and responsible antibiotic use. These findings can help improve hospital practices and protect both animal and public health.

## 1. Introduction

Intravenous catheters are commonly used in horses to provide sustained and secure access to the vein and to facilitate the administration of therapeutic or anesthetic drugs [1]. Thrombophlebitis is the most common frequently reported complication associated with catheterization and occurs most often in animals with systemic disease, including endotoxaemia, salmonellosis, and diarrhea [2,3]. Bacterial colonization of the catheter may increase the rate of occurrence of this complication and the associated morbidity [1,4,5].

Contamination of the injection ports allows microorganisms to travel endoluminally and can result in catheter-related bloodstream infections (CRBSIs) [6]. These bloodstream infections in critically ill patients may contribute to increased morbidity and mortality [7]. CRBSIs are diagnosed when the same microorganism is isolated from blood culture and the catheter tip in a patient with clinical signs of bacteremia [8]. Microorganism colonization of a catheter is not synonymous with CRBSIs and does not necessarily indicate local or systemic infection; however, it can be responsible for subsequent CRBSIs [9].

In human tertiary care hospitals, between 1.2% and 5.6% of catheter injection ports resulted positive for bacterial growth, and *Staphylococcus aureus* was the predominant organism isolated [6,10]. Veterinary studies on small animals found a positive catheter tip at microbiological culture in 10–24% of cases [11,12], and the most frequently isolated microorganisms belonged to the genus *Staphylococcus* [11]. A few studies have been performed on horses, in which the most common isolates were *Enterobacteriaceae*, *Micrococcus*, and *Staphylococcus* species [1,2].

While peripheral venous catheters are the most frequently used in humans and small animals, in horses, the placement of central venous catheters is more common by far, which are traditionally viewed as having a higher risk for infection and potentially severe consequences [11].

This study aimed to assess the incidence of bacterial catheter colonization in hospitalized equine patients with a variety of diseases.

## 2. Materials and Methods

### 2.1. Study Design and Population

This is a prospective clinical study performed over 2 years (2020–2021) on a cohort of sick equine patients admitted to the Veterinary Teaching Hospital “M. Modenato” of the University of Pisa. The study was performed during the regular course of the hospitalization of the animals and with the owner’s written consent. For all the patients enrolled, data on signalment, reason for admission to the hospital, diagnosis, duration of catheterization (hours), catheter complications (malfunctioning, infection site, thrombus), and antibiotic or corticosteroid treatment were collected. They were also divided based on age (newborns: <21 days; young animals: 21 days–1 year; adult: >1 year) and classified as survivors and non-survivors.

### 2.2. Catheter Placement and Management

Two types of catheters were used: a G14 polytetrafluoroethylene of 5 cm (PTFE) and a G14 polyurethane of 11 cm (PU). An animal was considered the same patient if another catheter was placed in the same vein or a contralateral vein within less than 2 weeks from the previous catheter. Both catheters were placed as previously described [13,14], in the jugular vein after aseptic preparation of the skin. Non-sterile disposable gloves were used for the PTFE placement, while PU was placed with sterile surgical gloves. The catheters received a locking solution with sodium chloride and were sutured in place and closed with a cap with no extension set. In foals, the catheter was covered with gauzes and an adhesive elastic bandage. Both catheters were inspected at least twice daily for swelling, pain, and venous ingurgitation, and flushed with heparinized saline every 6 h. Indications for catheter removal were death or euthanasia, evidence of local infection at the catheter site, or end of the intravenous treatment.

### 2.3. Sample Collection, Microbiological Processing, and Bacterial Identification

All catheters were removed using sterile gloves after skin disinfection with 10% povidone-iodine and alcohol and allowed to dry, and the catheter was removed through the clean field. A 2 cm distal segment was cut with sterile scissors and collected into sterile tubes. All samples were immediately sent under refrigerated conditions to the Laboratory of Infectious Diseases of the Department of Veterinary Sciences, University of Pisa for processing.

All samples were maintained at 4 °C and analyzed within 4 h from arrival to the laboratory. Bacteriological contamination of non-permanent venous catheters was assessed as previously described by other authors, with some modifications [15,16]. Briefly, 1 mL of sterile saline solution was added in the tube and catheter tip was washed by vigorously shaking and vortexing for 1 min. A loopful of the liquid was streaked on 2 plates of 5% Blood Agar (Thermo Fisher Diagnostics, Milan, Italy) and subsequently incubated at 37 °C in aerobic and anaerobic conditions for up to 10 days. Furthermore, 0.5 mL of the liquid was inoculated in 5 mL of Brain Heart Infusion broth (Thermo Fisher Diagnostics, Milan, Italy) and incubated at 37 °C up to 10 days. Brain Heart Infusion cultures were checked daily and if they turned positive (development of turbidity), they were sub-cultured on Blood Agar plates and incubated as previously described. Blood Agar plates were checked daily for bacterial growth. From positive plates, 1 to 3 colonies different in size, color, and shape were selected and sub-cultured on new Blood Agar plates in order to obtain pure cultures to be characterized.

Isolated bacteria were identified using standard laboratory techniques. Briefly, isolates were subjected to Gram staining and oxidase and catalase tests. Subsequently, they were sub-cultured on selective and differential media to be identified at least to the genus level. In particular, for Gram-positive isolates, the following media were used: Bacillus Cereus Agar (MYO) (Biolife, Milan, Italy), Mannitol Salt Agar (Thermo Fisher Diagnostics, Milan, Italy), Kanamycin Esculin Azide Agar (Thermo Fisher Diagnostics, Milan, Italy). While for Gram-negative bacteria, the following media were used: Violet Red Bile Glucose Agar (Thermo Fisher Diagnostics, Milan, Italy), Tryptone Bile X-Gluc Agar (Thermo Fisher Diagnostics, Milan, Italy), and Pseudomonas CFC Agar (Thermo Fisher Diagnostics, Milan, Italy). Isolates were identified at species level, if possible, using API tests (bioMérieux SA, Marcy l’Etoile, France), particularly API Staph, API Strep, API E, and API NE, following the manufacturer’s instructions.

If bacterial growth was present, catheters were considered “colonized.”

### 2.4. Statistical Analysis

The data collected were organized using Microsoft Excel for Mac (Microsoft Corp., Seattle, WA, USA) and analyzed using SPSS 27.0 statistical software for Mac (IBM, Armonk, NY, USA).

The normality of the distribution of quantitative variables was assessed using the Shapiro–Wilk test, and it was found that none of the variables followed a normal distribution. Descriptive statistics were reported separately for the venous catheter contamination status. The median, 25%, and 75% percentile values were used as quantitative variables, while frequency and percentage were used as qualitative variables. To determine the parameters correlated with the presence or absence of catheter contamination, a multivariate logistic regression model was developed. Initially, a univariate analysis was performed, and variables with a *p*-value less than 0.1 were considered potential independent variables for the regression model. Quantitative variables were analyzed using the Mann–Whitney U test, while qualitative variables were assessed using the Chi-Square test. Spearman’s correlation index was used to test the collinearity of these variables, and variables with an R-value exceeding 0.6 were considered collinear. The final multivariate logistic regression model was selected based on the highest pseudo-R-square value, and variables were considered to be correlated with venous catheter contamination if the *p*-value was less than 0.05.

## 3. Results

### 3.1. Study Population and Catheter Usage

In total, 58 catheters were collected for culture from 52 patients of different breeds, age, diagnosis, and treatments. The catheters were 32/58 (55.2%) PTFE and 26/58 (44.8%) PU. A detailed overview of the patients who underwent two catheterizations, as well as the age and breed distribution of the study population, is presented in Table 1.

### 3.2. Clinical Findings and Treatments

A total of 5 out of 52 patients (9.6%) developed thrombophlebitis of the catheterized vein during their hospitalization. Three of them were adults with a PTFE catheter, and two were foals with a PU catheter. Then, 40 out of 52 (76.9%) patients were treated with intravenous antimicrobials and 13/52 (25%) with intravenous corticosteroids. There were 49/52 (94.2%) survivors and 6/52 (11.5%) patients who died or were euthanized.

### 3.3. Bacterial Colonization and Microbiological Results

A total of 38 positive catheters (65.5%) were detected (24/38 PTFE and 14/38 PU). In total, 13 out of 38 samples (34.2%) were positive through the direct culture of the sample and after enrichment (8 PTFE and 5 PU), while an additional 24/38 (65.1%) turned positive only after Brain Heart Infusion enrichment (15 PFTE and 9 PU). In 1/38 (2.6%) positive catheters, different bacteria were isolated from direct culture and enrichment (*Staphylococcus* spp. by direct culture and *Bacillus* spp. after enrichment), while in 12/13 cases, the two isolation protocols employed allowed the isolation of bacteria belonging to the same genus. In 3/38 (7.9%) of the positive samples, two different bacteria were isolated from the same catheter after enrichment. A total of 43 bacterial strains were isolated from the 38 positive samples; 9/43 isolates were Gram-negative; and 34/43 isolates were Gram-positive. In particular, the isolated Gram-positive bacteria belonged to the genera *Staphylococcus* spp. (19/43), *Bacillus* spp. (9/43), and *Enterococcus* spp. (4/43). Among Gram-negative strains, 7/43 belonged to the *Enterobacteriaceae* family and 2/43 to the *Pseudomonas* genus. Of the 38 positive catheters, 24/38 were associated with an antimicrobial treatment and 6/38 with corticosteroids treatment. The results of the cultured examination of each catheter from each patient, reporting outcomes after direct culture and/or enrichment, are reported in the Supplementary Materials (Table S1).

### 3.4. Statistical Analysis and Risk Factors

The univariate analysis identified the type of catheters, the time of the catheter’s permanence, the treatment with or without antibiotics, corticosteroids, and patients with thrombophlebitis as parameters to insert in the logistic regression model (*p*-value < 0.1) (Table 2). However, in the final multivariable logistic regression model, only the use of antibiotics was found to be correlated with catheter contamination, with a *p*-value of 0.005 (Table 3). The odds ratio showed that horses not treated with antibiotics had a 10.9 (95% CI: 7.3–15.9) times higher likelihood of developing catheter contamination compared to those treated with antibiotics. No other variables were identified as being associated with catheter contamination in the study.

## 4. Discussion

In this prospective study, 65.5% of catheters demonstrated colonization by one or more microorganisms. This percentage is significantly higher than previously reported rates for humans (0.7–2.2%) [10], small animals (10.4–22%) [11,12,17], and horses (7–13%) [1,2]. However, Ettlinger et al. (1992) reported a similar prevalence of contamination (50–73%) in catheters removed from equine patients [18]. These differences can largely be attributed to a combination of factors, including variations in the placement, ongoing management, and timely removal of catheters; differences in microbiological culture techniques and laboratory protocols; environmental and institutional conditions; and the overall severity and complexity of systemic illness among the patient populations included in the different studies.

The contamination of catheters and subsequent local and systemic infections occurs via several mechanisms, the most common of which is migration of microorganisms from the skin of healthcare workers or patients at the catheter insertion site or hub [11]. Although the role of catheter contamination in subsequent local or systemic infection is unknown, colonization by microorganisms, particularly biofilm-producing microbes, has the potential to result in serious bloodstream infections [7]. In horses, catheter contamination constitutes one of the risk factors for the development of thrombophlebitis [4]. However, no significant relationship between the bacterial colonization of catheters and the development of thrombophlebitis has been previously reported in this species [1,2]. Despite not using ultrasonography to monitor for subclinical thrombophlebitis in this study, and the small number of patients who developed this complication, all cases with thrombophlebitis showed a positive catheter culture.

The most frequently isolated bacteria in the current study belonged to the genus *Staphylococcus*, which is consistent with what has already been reported both in human and veterinary studies [11,19]. The high prevalence of *Staphylococcus* spp. colonization in both human and veterinary research is explained by its commensal residence on the skin and its membership to endemic hospital flora [20].

Our study found no association between wearing sterile or non-sterile gloves and catheter contamination, which aligns with previous results described by Saloojee and colleagues [21], who suggested that, if there has been no contact with an adequately scrubbed insertion site prior to cannulation and the inserter’s hands are appropriately washed, gloves are not necessary [21].

Furthermore, we did not find catheter dwell time to be a risk factor for colonization. The previous literature regarding catheter dwell time has been controversial [11,12]. However, in this and in previous studies, it is impossible to compare PTFE short-term catheters with PU long-term catheters since dwell time is closely related to the thrombogenicity of the catheter.

The only risk factor for catheter contamination that we could identify was the absence of systemic antimicrobial treatment. This finding is compatible with what has been described in the past for human patients, in which antimicrobial-impregnated catheters were associated with a significantly lower colonization rate [22]. However, the use of antimicrobials as a preventative measure to avoid contamination is not a valid strategy as this has been demonstrated to lead to antimicrobial resistance [23]. In small animals, no association between the catheter contamination and antimicrobials or corticosteroids administration was found; however, in the study of Matula et al. (2023) [11], the use of systemic antimicrobials was associated with the antibiotic resistance of the bacteria isolated [11]. Furthermore, in horses, the administration of non-steroidal anti-inflammatory drugs through the catheters was shown to protect against subclinical vascular changes but no significant relationship between the catheter’s contamination and the development of thrombophlebitis was observed [1].

Due to the variety of diseases of the patients included in this study and the relatively small number of cases, we could not evaluate the association between catheter contamination and presence of systemic inflammatory response syndrome, which could be a possible risk factor and thus constitutes the first limitation of this investigation. Furthermore, in a previous study, the frequency of catheter colonization was found to be associated with the experience of the clinician with up to a two-fold increase in the frequency of colonization when the catheters were placed by less experienced individuals [24]. Unfortunately, we did not record the clinician who applied each catheter or the difficulties faced during catheterization or the animal reaction, which were also found to be associated with the risk of contamination. An additional limitation of this study is the lack of a quantitative or semi-quantitative culture method to evaluate the degree of catheter contamination and the lack of antibiogram testing to determine the sensitivity of the cultured bacteria. Furthermore, no blood culture was performed, from neither the catheter lavage nor peripheral site; however, CRBSIs were not the objective of this research.

In conclusion, we report a notably high rate of catheter contamination compared with what has been previously described in horses [1,2]. Most of those isolated were Gram-positive, as already described by others [11,12,17]. Although the microbial colonization of intravenous devices may not be clinically relevant for each patient, the high contamination rate is concerning due to the risks related to antimicrobial resistance and zoonotic potential. Therefore, further studies are warranted to assess the association between catheter contamination, bloodstream infections, systemic signs of infection, and antimicrobial sensitivity.

## Figures and Tables

**Table 1:** Summary of the six patients who received two catheterizations, along with the age and breed distribution of the 52 enrolled patients.

Category	Details
Patients with 2 Catheters (*n* = 6)	- 3 Adults: 2 jennies, 1 trotter mare (PTFE both times) - 2 Newborns: 1 Standardbred colt (PTFE and PU), 1 Crossbred filly (PU and PU) - 1 Young: Female Standardbred (PTFE and PU)
Age Distribution (*n* = 52)	- Foals: 16 (11 colts, 5 fillies) - Young: 7 (6 males, 1 female) - Adults: 29 (16 females, 4 geldings, 9 stallions)
Breed Distribution (*n* = 52)	- Standardbred: 13 - Warmbloods: 9 - Donkeys: 9 - American breeds (Appaloosa and Quarter Horse): 6 - Mules: 4 - Draft horses: 3 - Ponies: 4 - Hotblood (Thoroughbred and Arabian): 3 - Crossbred: 1

Abbreviations: PU, polyurethane; PTFE, polytetrafluoroethylene.

**Table 2:** Univariate analysis of the presence or absence of catheter contamination.

Variable	Negative Culture	Positive Culture	*p*-Value
Type of catheter			
PTFE	8 (40.0%)	24 (63.2%)	0.092
PU	12 (60.0%)	14 (36.8%)	
Antibiotic treatment			
Treatment without antibiotics	1 (5.0%)	14 (36.8%)	0.008
Treatment with antibiotics	19 (95.0%)	24 (63.2%)	
Corticosteroid treatment			
Treatment without corticosteroids	13 (65.0%)	32 (84.2%)	0.095
Treatment with corticosteroids	7 (35.0%)	6 (15.8%)	
Thrombophlebitis			
Absence of thrombophlebitis	20 (100.0%)	33 (86.8%)	0.090
Presence of thrombophlebitis	0 (0.0%)	5 (13.2%)	
Sepsis			
Absence of sepsis	18 (90.0%)	32 (84.2%)	0.543
Presence of sepsis	2 (10.0%)	6 (15.8%)	
Outcome			
Survived	18 (90.0%)	34 (89.5%)	0.950
Not survived	2 (10.0%)	4 (10.5%)	
Time of permanence (hours)	96 (24–144)	36 (7.5–76.25)	0.049

**Table 3:** Results of the multivariate regression model.

Variables	Category	*p*-Value	OR	CI 95%
Antibiotics treatment	Treatment without antibiotics	0.005	10.9	7.3–15.9
	Treatment with antibiotics	-	1.0	-

Abbreviations: OR, odds ratio; Cl, confidence interval. Note: Treatment with antibiotics was used as the reference category for comparison.

## Data Availability

Data presented in this study are available on request from the corresponding author.

## Supplementary Materials

The following supporting information can be downloaded at https://www.mdpi.com/article/10.3390/vetsci12090788/s1, Table S1: Results of cultured examination of each catheter from each patient, reporting outcomes after direct culture and/or enrichment.

## Abbreviations

The following abbreviations are used in this manuscript:

CRBSIs	Catheter-Related Bloodstream Infections;
PTFE	Polytetrafluoroethylene;
PU	Polyurethane.
