# Supplementary material for: Incidence of Bacterial Colonization of Intravenous Non-Permanent Venous Catheters in Hospitalized Equine Patients
Source: Vet Sci. 2025 Aug 22;12(9):788. doi: 10.3390/vetsci12090788 (PMC12474212; doi:10.3390/vetsci12090788)
Supplement: Supplementary file 1 [file vetsci-12-00788-s001.zip › vetsci-3767647-supplementary.pdf]

Table S1. Results of cultured examination of each catheter from each patient, reporting outcomes after direct culture and/or enrichment.

Catheter	Breed	Age	Sex	Types of Catheters	Culture	Bacteria
1	Saddle Horse	1 day old	M	PU	Positive	*Staphylococcus warneri*
2	Donkey	13 years old	F	PTFE	Positive	*Staphylococcus epidermidis*
3	Donkey	13 years old	F	PTFE	Positive	*Staphylococcus epidermidis*
4	Donkey	16 years old	F	PTFE	Positive	*Bacillus* spp.
5	Donkey	16 years old	F	PTFE	Positive	*Bacillus* spp.
6	Trotter	15 years old	F	PTFE	Positive	*Bacillus* spp.
7	Trotter	18 years old	F	PTFE	Positive	*Bacillus* spp., *Staphylococcus aureus*
8	Trotter	19 years old	F	PTFE	Positive	*Staphylococcus sciuri*
9	Donkey	13 years old	F	PTFE	Positive	*Staphylococcus epidermidis*
10	Saddle Horse	1 day old	M	PU	Negative	
11	Draft Horse	8 years old	M	PTFE	Positive	*Staphylococcus aureus*
12	Donkey	15 years old	M	PTFE	Positive	*Staphylococcus warneri*
13	Draft Horse	16 years old	M	PTFE	Negative	
14	Draft Horse	15 years old	M	PTFE	Positive	*Bacillus* spp., *Klebsiella pneumoniae*
15	Trotter	5 days old	F	PU	Negative	
16	Trotter	8 years old	F	PTFE	Negative	
17	Trotter	8 years old	F	PTFE	Positive	*Staphylococcus epidermidis*
18	Saddle Horse	60 days old	M	PTFE	Positive	*Enterococcus faecalis*
19	Arabian Horse	1 day old	M	PU	Negative	
20	Trotter	30 days old	M	PTFE	Negative	
21	Trotter	30 days old	M	PU	Positive	*Enterobacter cloacae*
22	Quarter Horse	5 months old	M	PU	Negative	
23	Arabian Horse	20 years old	F	PTFE	Negative	
24	Saddle Horse	19 years old	G	PTFE	Positive	*Staphylococcus epidermidis*
25	Trotter	1 year old	F	PU	Negative	
26	Saddle Horse	2 days old	M	PU	Negative	
27	Mule	2 years old	M	PTFE	Negative	
28	Quarter Horse	17 years old	F	PTFE	Positive	*Staphylococcus sciuri*
29	Saddle Horse	3 days old	M	PU	Positive	*Bacillus* spp.
30	Saddle Horse	1 day old	M	PU	Positive	*Bacillus* spp.
31	Donkey	22 years old	M	PU	Positive	*Staphylococcus epidermidis*
32	Pony	10 years old	F	PU	Positive	*Enterobacter cloacae*
33	Mule	15 days old	M	PTFE	Positive	*Bacillus* spp., *Staphylococcus warneri*
34	Mule	5 days old	M	PU	Positive	*Staphylococcus epidermidis*
35	Appaloosa	4 years old	G	PU	Positive	*Staphylococcus aureus*
36	Quarter Horse	8 months old	M	PU	Negative	
37	Quarter Horse	16 years old	F	PTFE	Positive	*Staphylococcus epidermidis*
38	Donkey	8 years old	M	PTFE	Negative	
39	Crossbred	2 days old	F	PTFE	Positive	*Enterococcus faecalis*
40	Crossbred	6 days old	F	PU	Positive	*Enterococcus cloacae, Enterococcus faecium*
41	Pony	2 years old	M	PU	Negative	
42	Pony	4 years old	F	PTFE	Negative	
43	Pony	5 days old	M	PU	Negative	
44	Trotter	1 day old	M	PU	Positive	*E. coli*
45	Trotter	4 days old	M	PU	Positive	*Enterobacter cloacae*
46	Trotter	4 days old	M	PU	Positive	*Enterococcus durans*
47	Donkey	45 days old	F	PU	Negative	
48	Trotter	4 days old	F	PTFE	Positive	*Staphylococcus epidermidis*
49	Trotter	90 days old	M	PU	Positive	*Enterobacter cloacae*
50	Mule	5 days old	F	PU	Negative	
51	Quarter Horse	13 years old	G	PU	Negative	
52	Saddle Horse	16 years old	G	PTFE	Positive	*Pseudomonas aeruginosa*
53	Donkey	16 years old	F	PTFE	Positive	*Klebsiella pneumoniae*
54	Donkey	4 years old	M	PTFE	Positive	*Enterococcus faecalis, Staphylococcus sciuri*

55	Trotter	15 years old	F	PTFE	Positive	*Bacillus* spp.
56	Thoroughbred	5 days old	F	PTFE	Positive	*Pseudomonas aeruginosa*
57	Trotter	9 years old	F	PTFE	Negative	
58	Saddle Horse	19 years old	G	PU	Positive	*Staphylococcus epidermidis*

Abbreviations: M, male; F, female; G, gelding; PU, polyurethane; PTFE, polytetrafluoroethylene.
